# Machine learning-enabled quantitative ultrasound techniques for tissue differentiation

**DOI:** 10.1007/s10396-022-01230-6

**Published:** 2022-07-15

**Authors:** Hannah Thomson, Shufan Yang, Sandy Cochran

**Affiliations:** 1 Centre for Medical and Industrial Ultrasonics, University of Glasgow, University Avenue, Glasgow, UK; 2 School of Computing, Edinburgh Napier University, Merchiston Campus, Edinburgh, UK

**Keywords:** Quantitative ultrasound, Ultrasound phantoms, Tissue characterisation, Parametric imaging, Binary classifier, Machine learning

## Abstract

**Purpose:**

Quantitative ultrasound (QUS) infers properties about tissue microstructure from backscattered radio-frequency ultrasound data. This paper describes how to implement the most practical QUS parameters using an ultrasound research system for tissue differentiation.

**Methods:**

This study first validated chicken liver and gizzard muscle as suitable acoustic phantoms for human brain and brain tumour tissues via measurement of the speed of sound and acoustic attenuation. A total of thirteen QUS parameters were estimated from twelve samples, each using data obtained with a transducer with a frequency of 5–11 MHz. Spectral parameters, i.e., effective scatterer diameter and acoustic concentration, were calculated from the backscattered power spectrum of the tissue, and echo envelope statistics were estimated by modelling the scattering inside the tissue as a homodyned K-distribution, yielding the scatterer clustering parameter *α* and the structure parameter *κ*. Standard deviation and higher-order moments were calculated from the echogenicity value assigned in conventional B-mode images.

**Results:**

The k-nearest neighbours algorithm was used to combine those parameters, which achieved 94.5% accuracy and 0.933 F1-score.

**Conclusion:**

We were able to generate classification parametric images in near-real-time speed as a potential diagnostic tool in the operating room for the possible use for human brain tissue characterisation.

## Introduction

Ultrasound has significant advantages for intraoperative imaging including real-time capability, portability, and ease of use in combination with other technologies such as navigation systems in the neurosurgical theatre [1]. However, ultrasound applied conventionally also has limitations, which may be the reason the technique has not been adopted by all neurosurgeons. It requires specific experience to obtain optimum image quality as it is qualitative in nature and hence relies strongly on clinical interpretation. Furthermore, there exists an inherent trade-off between image resolution and penetration depth.

Quantitative ultrasound (QUS) is a technique that infers properties of tissue microstructure based on analysis of radio-frequency (RF) ultrasound data before image processing. The speckled pattern in B-mode images arises from scattering that occurs when ultrasound encounters acoustic inhomogeneities with dimensions similar to or smaller than its wavelength—around 200 µm in brain tissue at a typical medical ultrasound imaging frequency of 7.5 MHz. Compelling evidence has been published on the ability of QUS to detect cancerous regions in several soft tissues. In particular, it has benefits in detection of ocular [2] and prostate [3, 4] cancers, metastases in lymph nodes [5, 6] and in classification of breast masses [7], with recent studies adopting multiparametric and machine-learning approaches [8].

Despite its success, the use of QUS in neuro-oncology has not yet been explored fully, especially in glioma surgery. One study showed that measurements of acoustic attenuation and backscatter coefficient could be used to differentiate healthy brain, oedema, and meningioma in vivo at 5 MHz [9]. Another instance of QUS in neural tissue looked at simple spectral parameters to measure murine response to radiotherapy through the skull and was found to be a reliable indicator of treatment [10]. However, a full QUS analysis for healthy and cancerous human brain tissue has yet to be done.

Fresh human samples of brain and brain tumour of an adequate size are difficult to obtain for logistical, safety, and ethical reasons. It can be difficult to obtain fresh samples of healthy tissue due to the rapid deterioration of brain tissue when removed from the body. Secondly, tumour tissue of an adequate size is difficult to access as the tumour is often ablated in small sections, and the tumour bulk should be retained for pathological research [11]. Even brain tissue banks, which offer frozen and fixed healthy brain tissue, often only provide small samples that are only suitable for clinical brain sciences, and not ultrasound research.

To improve the situation and increase the potential for use of ultrasound in the neurosurgical theatre, we propose a user-independent classification tool based on QUS techniques to classify phantoms of healthy brain tissue and brain tumours using chicken liver and gizzard muscle. First, we evaluated the suitability of chicken liver and gizzard to act as phantoms of brain and brain tumour via acoustic characterization. We found that the speed of sound and attenuation of the tissues were similar over the frequency range 1–10 MHz. A total of thirteen QUS parameters from the phantom of chicken liver and gizzard muscle were then estimated from 12 samples of each using data obtained with a medical ultrasound research system with a transducer with a frequency of 5–11 MHz. The obtained QUS parameters were used in training the K-nearest neighbour algorithm (KNN), which is described in the following section. The KNN can achieve high classification accuracy with nine parameters at a reduced processing time compared with the original thirteen parameters. The differentiated parametric images with a near-real-time processing speed can be used as a potential diagnostic tool in the operating room.

## Materials and methods

### Macroscopic acoustic properties

Twelve samples of fresh chicken liver and gizzard muscle were purchased from a local Halal butcher. The samples were cut into small slices of uniform, but varying thickness with a scalpel, and all measurements were made in a temperature-controlled environment at 20 °C. The density of the tissue was calculated by measuring the mass and estimating the volume via the Archimedes Principle. To calculate the speed of sound, c, the tissue was placed on the surface of a single-element 10-MHz transducer (unfocused immersion transducer, aperture 10 mm; Olympus Scientific Solutions Technologies, MA, USA). A holder was designed to surround the transducer, supporting the tissue to sit parallel to its surface.

The transducer was connected to an ultrasonic pulser-receiver (DPR300; JSR-Imaginant, NY, USA) operating in pulse-echo mode, and the receiver output was connected to an oscilloscope (InfiniiVision 2000 X-Series, 200 MHz; Keysight Technologies, CA, USA). The time, ∆t, between successive echoes was measured using the oscilloscope measuring function, and the speed of sound in the tissue was calculated according to *c* = 2*d*/∆*t*, where *d* is the sample thickness. This procedure was repeated for 12 samples of both liver and gizzard, and the average value was taken.

Calculation of attenuation was based on the transmission loss method [12]. A needle hydrophone (0.2-mm diameter, 8-dB preamplifier; Precision Acoustics Ltd., Dorset, UK) was positioned at the natural focal point of the transducer in a tank of degassed water. An empty sample holder was placed in the acoustic path, and measurements were taken for the reference voltage, $V_{{{\text{water}}}}$. Next, a tissue sample of known thickness was encased in agar to hold it securely in the sample holder, and a reduced voltage was recorded by the hydrophone, $V_{{{\text{sample}}}}$. Attenuation due to the insertion of the tissue sample at a single frequency can be calculated from:

1 $$\alpha_{{{\text{sample}}}} = - \frac{20}{d}\log_{10} \left( {\frac{{V_{{{\text{sample}}}} }}{{V_{{{\text{water}}}} }}} \right) + \alpha_{{{\text{water}}}} ,$$

where *α*_sample_ is the attenuation of the test sample, *d* is the sample thickness in cm, and *α*_water_ is the attenuation of water in dB/cm at the frequency of interest. This procedure was carried out using immersion transducers designed to operate at three different frequencies: 1, 5, and 10 MHz (all unfocused immersion transducers; Olympus Scientific Solutions Technologies, MA, USA). Measurements were repeated five times for each of the twelve samples at each frequency, and the attenuation coefficient was estimated as the gradient of a best-fit straight line through the average values found for each frequency.

### Quantitative ultrasound (QUS)

As an alternative to conventional B-mode imaging, QUS has been studied in a variety of applications over the last 50 years [13]. Scattering arises from small spatial changes in the acoustic impedance, i.e., the product of density and speed of sound, around a bulk value. If the wavelength of ultrasound is larger than or of the same order as the impedance deviation then ultrasound energy will be scattered in directions dependent on the size, shape, and orientation of the scatterer relative to the incident wave [14]. Some of the energy will be scattered back to the transducer, and the signal received by the transducer, prior to any processing, is referred to as the RF data.

#### Spectral methods

The frequency dependence of the RF data can be used on its own to characterise tissue. This was demonstrated in pioneering studies [15] via measurements of the backscatter coefficient, i.e., the amount of ultrasound energy scattered back at 180° and received by the transmitting transducer [13]. To understand the physical basis of the frequency dependence, mathematical models describing the size, shape, and distribution of scatterers within tissue were subsequently developed. The one that has shown the most accuracy and been used most often is the spherical Gaussian model developed by Lizzi et al. [16, 17]. It relates the backscattered power spectrum from tissue regions to two key parameters describing tissue microstructure: the effective scatterer diameter, $2a_{{{\text{eff}}}}$, and the acoustic concentration, defined as $\rho z_{{{\text{var}}}}^{2}$ where *ρ* is the effective concentration of scatterers per mm3 and $z_{{{\text{var}}}}^{2}$ is the relative acoustic impedance difference between the scatterers and the surrounding material. Assuming a Gaussian form factor, the theoretical backscatter coefficient can be written as:

2 $$W\left( f \right) = \frac{{\pi^{4} }}{{36c^{4} }}f^{4} \rho z_{{{\text{var}}}}^{2} exp\left( { - \frac{{a_{{{\text{eff}}}} }}{{\left( {12\sqrt {2\pi } } \right)^{1/3} }}} \right)^{2} \left( {\frac{{2\pi f^{2} }}{c}} \right),$$

where *c* is the speed of sound. In order to solve for the effective scatterer diameter and acoustic concentration, the transducer geometry and frequency dependence of the instrumentation must be known, the latter requiring calibration through a reference measurement. The scatterer size estimate, in particular, has shown success in characterising cancerous tissue. Oelze et al*.* made estimates from suspected cancerous lymph nodes and found that metastatic nodes had significantly higher $a_{{{\text{eff}}}}$ than cancer-free nodes. Similar results were observed in rat mammary tumours in vivo, where estimates of scatterer size inside the tumour region were 44% larger than outside [18].

#### Statistical methods

Statistical methods, called echo envelope techniques, were developed in parallel with spectral methods, with the aim to model the distribution of echo amplitudes from tissue scattering regions. The echo envelope signal is obtained from the magnitude of the Hilbert transform of the RF data from a tissue scattering region [19]. This signal is then modelled mathematically to yield parameters to describe the organisation structure, density, and concentration of scattering components [20]. Various models have been used in the literature to describe the echo envelope statistics, the most fundamental being the Rayleigh distribution, which describes randomly located and densely packed scatterers [21]. Others include, but are not limited to, the Rice distribution, the homodyned K-distribution, the K-distribution, and the Nakagami distribution [22]. The Nakagami distribution is most commonly used for tissue characterisation and is an approximation of the homodyned K-distribution.

In the present study, the homodyned K-distribution was used to model the echo envelope statistics from tissue scattering regions as it represents a general scattering distribution with a varying density of random scatterers with or without a coherent component [23].

Solving for the model parameters of the homodyned K-distribution can provide additional statistical QUS parameters which can be implemented into the binary classification.

### QUS data acquisition

**Fig. 1:**
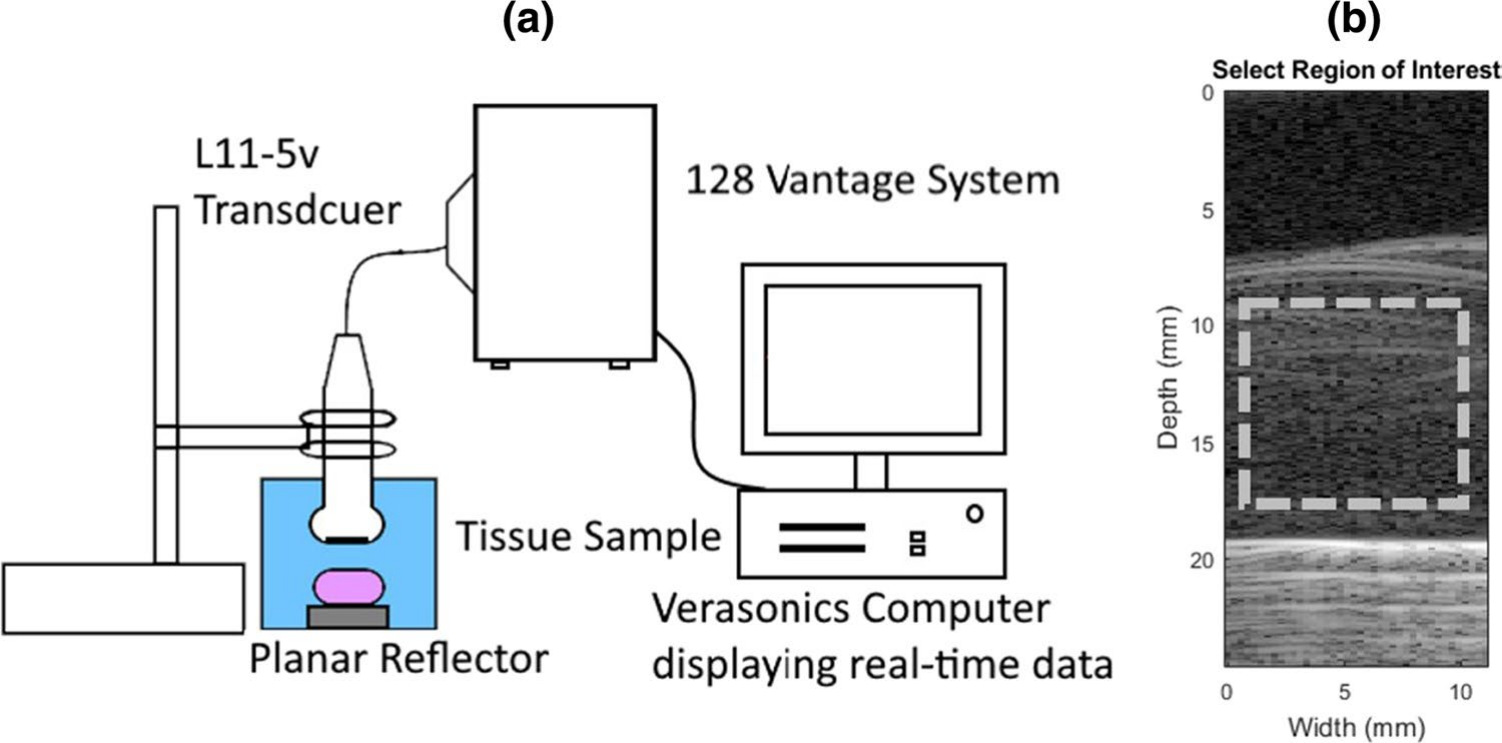
Experimental set-up of data acquisition from tissue using Verasonics Vantage system

For the QUS study, the experimental set-up consisted of the tissue submerged in water and placed on a quartz flat below the centre of a linear array probe (Verasonics, Inc., WA, USA) with a frequency of 5–11 MHz, connected to a Vantage 128 ultrasound research system (Verasonics, Inc., WA, USA) in a temperature-controlled room at 20 °C, as shown in Fig. 1a. The ultrasound research system was programmed to provide a plane wave by excitation of all elements simultaneously without beam forming, and then to acquire and store the RF signal received by each element. Each signal was sampled at 31.25 MHz, and the resulting RF data were stored in the host computer for offline processing. For processing, the RF data sets were loaded into a custom MATLAB (The Mathworks, Cambridge, UK) framework, allowing the user to select a region of interest (ROI), as in Fig. 1b.

**Fig. 2:**
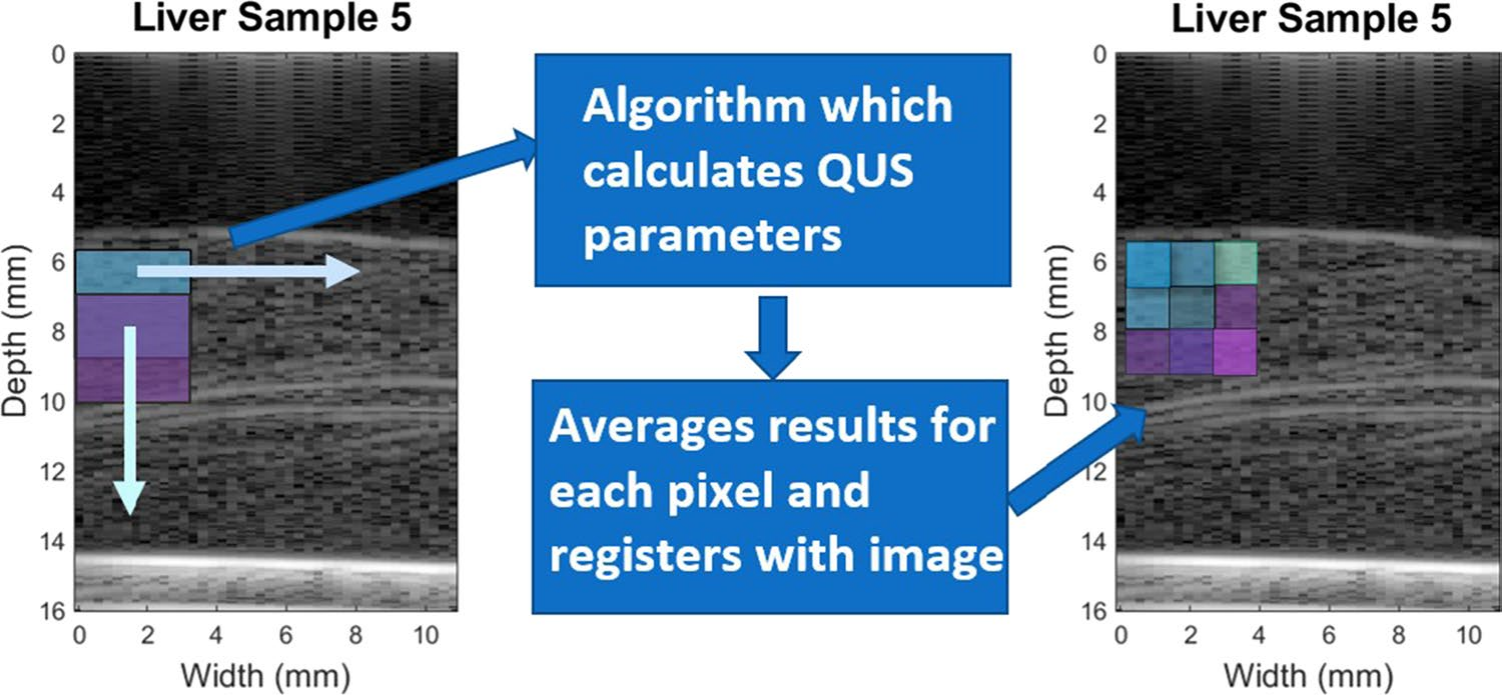
Diagram showing the sliding window technique for QUS parametric image formation

The ROI was divided into windows of 3 mm × 3 mm, corresponding to at least 10 wavelengths in the axial direction, and 12 transducer elements in the lateral direction. The windowed RF data were input into the algorithm, which calculated the QUS parameters for that specific window location. Details of the calculation of parameters from the RF data are provided in the following sections of this paper. To increase the resolution of the parametric image, a sliding window was used with 66% overlap. This allowed parameters to be calculated for the larger 3 mm × 3 mm window, but the resulting parametric image had a pixel value for each 1 mm × 1 mm region, as shown in Fig. 2, albeit with some correlation between values in successive pixels.

### QUS analysis

#### Scatterer size and acoustic concentration

Estimation of scatterer size and acoustic concentration began by measuring a calibration power spectrum for each element in the array from a quartz flat placed at the same axial distance, *R*, as the centre of the tissue ROI. The power spectrum was defined as the squared magnitude of the gated time signal; in this study, a Hamming window was used as the gating function:

3 $$W_{{{\text{ref}}}} \left( f \right) = \left| {{\text{FFT}}\left[ {V_{{{\text{ref}}}} \left( {t,L} \right)*{\text{Ham}}\left( {t,L} \right)} \right]} \right|^{2}$$

where *L* is the length of the ROI and $V_{{{\text{ref}}}}$ is the reference signal amplitude. The power spectrum from each tissue ROI, $W_{t} \left( f \right)$, was measured in a similar way, and the experimental backscatter coefficient for each ROI was estimated using [24] with 12 active elements:

4 $$\sigma_{b} \left( f \right) = \frac{{6\left( {R + \frac{L}{2}} \right)^{2} d^{2} }}{{DLw^{2} h}}\frac{{\gamma^{2} }}{4}A\left( {f,L} \right)\frac{1}{N}\mathop \sum \limits_{i = 1}^{N} \frac{{W_{{{\text{tis}}}} \left( f \right)}}{{W_{{{\text{ref}}}} \left( f \right)}}$$

where *R* is the axial distance from the transducer to the centre of the ROI, *D* is the active area of the 12 transducer elements, *L* is the gate length, $\gamma$ is the reflectivity coefficient of a water and quartz interface, and $A\left( {f,L} \right)$ is an attenuation compensation function for a gated signal [25]. Note that, following convention, all lengths were defined in mm. The scatterer size was estimated from the backscatter coefficient by expressing it in decibel form and applying linear regression to solve for slope and intercept values [26]. The size could then be compared to the theoretical description of the backscatter coefficient, with parameters estimated as follows:

5 $$- 10\log_{10} \sigma b\left( f \right) = M\left( {a^{2} } \right)f + I\left( {a,CQ^{2} } \right)$$

6 $$M = 1.85 - 265a^{2}$$

7 $$I = 58.3 + 114\log \left( a \right) + 14.3\left[ {\log \left( a \right)} \right]^{2} + \log \left( {CQ^{2} } \right).$$

#### Homodyned K-distribution parameters

Using the same sliding window technique, homodyned K-distribution parameters were estimated for each window within the ROI. This requires the estimation of the model parameters $\left( {\epsilon , \sigma^{2} ,\alpha } \right)$, for which several methods exist. The method based on the mean and two log moments of intensity of the echo envelope signal was chosen in this study due to its high accuracy and speed [23]. First, the echo intensity distribution for a tissue region was found by taking the square of the magnitude of the Hilbert transform of the RF data. The *X* and *U* statistics were then calculated experimentally from this region by calculating the mean of the echo envelope signal and using the expressions below:

8 $$U : = \overline{\log I} - \log \overline{I} = U_{HK} \left( {\epsilon , \sigma^{2} ,\alpha } \right)$$

9 $$X: = \overline{I\log I} /\overline{I} - \overline{\log I} = X_{HK} \left( {\epsilon , \sigma^{2} ,\alpha } \right),$$

where $\overline{I}$ indicates the expectation value of the variable *I*. Next, by assuming the echo envelope signal amplitude, *A*, is distributed according to the homodyned K-distribution [23], the algebraic expressions for the *X* and *U* statistics could be obtained via $\overline{I}$, log(*I*), and *I* log(*I*), where *I* = *A*^2^.

The two nonlinear equations for *X* and *U* were solved for the model parameters $\left( {\epsilon , \sigma^{2} ,\alpha } \right)$ through a series of algorithms described by Destrempes et al. [23]. Two functions of the model parameters are invariant under scaling of the mean value, so they can be used as QUS parameters for tissue characterization: the scatterer clustering parameter, *α*, and the structure parameter $k = E^{2} /\left( {2\sigma^{2} \alpha } \right)$. It is worth noting that these parameters are particularly useful for tissue characterisation as they are system independent, so they do not depend on the amplitude or frequency of the ultrasound system.

#### Echogenicity parameters

Further parameters can be estimated solely from the grey level or echogenicity values of the B-mode image produced by the ultrasound system. After log compression of the RF data, the image is displayed according to the dynamic range. In this study, a dynamic range of 80 dB was used, and the distribution of echogenicity values in a given ROI yielded the variance and higher order moments of each B-mode image from simple statistics.

### Machine learning based classification

The QUS parameters obtained in the previous section allow for easy implementation into a machine learning classifier. A summary of the 13 parameters used is shown in Table 1.

**Table 1:** Summary of QUS parameters used in machine learning training algorithm

Symbol	Description
*a*	Effective scatterer diameter
*CQ*^2^	Acoustic concentration
$\overline{E}$	Echogenicity value mean
$\overline{E}^{2}$	Echogenicity value variance
$\overline{E}^{3}$	Echogenicity value skewness
$\overline{E}^{4}$	Echogenicity value kurtosis
$\overline{E}^{6}$	Echogenicity value 6th moment
*X*	*X* statistic
*U*	*U* statistic
*E*^2^	Coherent signal power
*σ*^2^	Diffuse signal power
*α*	Scatter clustering parameter
*κ*	Structure parameter

The k-nearest neighbour algorithm was trained using *k* = 5 from several combinations of the parameters from the spectral, statistical, and echogenicity-based methods. The QUS pixel data from 10 of the 12 samples of each tissue type were randomly chosen and used as training data for the machine learning classifier. This resulted in a dataset T that included 824 sets of parameters for liver and 648 for gizzard, as liver was thicker on average. The two remaining samples were used to test the performance of the classifier.

## Results

### Acoustic characterisation

The results for density, speed of sound, and attenuation coefficient for liver and gizzard are summarised in Table 2.

**Table 2:** Acoustic characterisation of liver and gizzard

Tissue	Density (kg/m^3^)	Speed of sound (ms^−1^)	Attenuation coefficient (dB/cm/MHz)
Liver	1067 ± 23	1539 ± 85	0*.*66 ± 0*.*16
Gizzard	1051 ± 8	1510 ± 44	0*.*81 ± 0*.*18

**Fig. 3:**
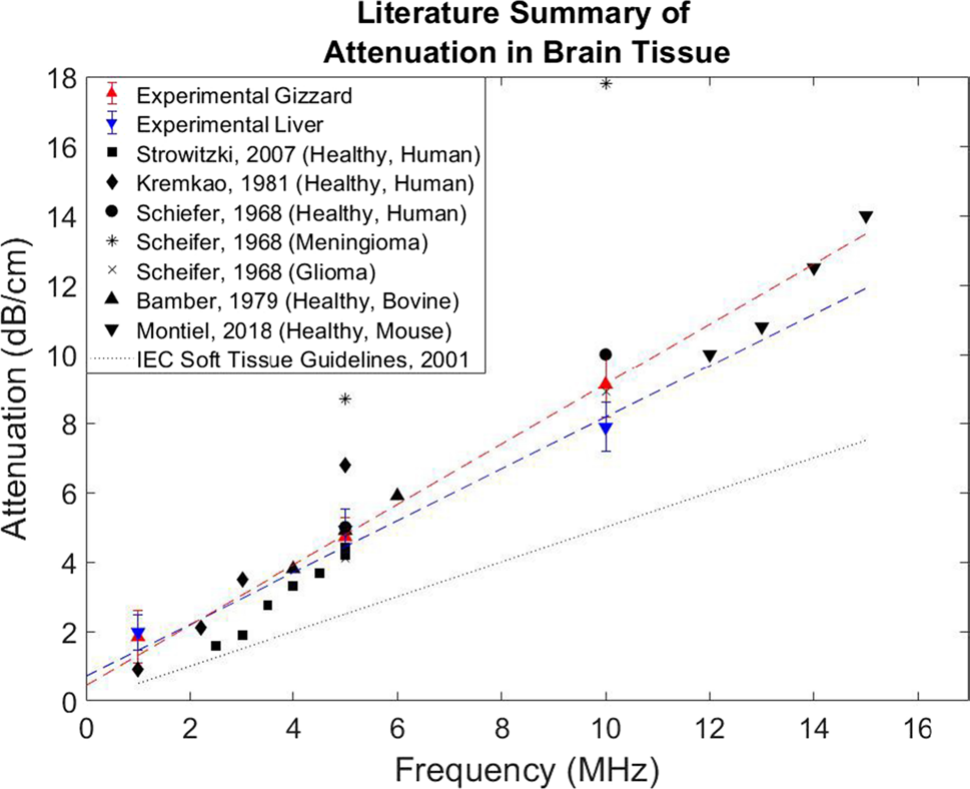
Comparison of attenuation values of healthy and cancerous brain tissue both in vivo and ex vivo. The dotted line shows soft tissue phantom standard guidelines of 0.5 dB/cm/MHz. The experimental results for liver and gizzard are shown with error bars as standard deviation of the mean at each frequency. The dependence of attenuation on frequency is assumed linear in this study

The speed of sound values agree with published values for healthy brain and glioblastoma. An attenuation value measured for each frequency, showing the variance on the mean, along with a summary of the literature values, are given in Fig. 3. The attenuation coefficients were determined to be 0.66 and 0.81 dB/cm/MHz for liver and gizzard, respectively. When compared to the value for healthy brain, the attenuation value for liver agrees well over the frequency range of interest. Values in the literature manifest significant variation and, importantly, the two reported instances of ex vivo cancerous brain tissue show attenuation in glioma to be only slightly above the values for healthy brain, whereas the meningioma shows a larger difference.

We thus conclude that our results are evidence that liver and gizzard are reasonable phantoms for brain and brain tumour over the present frequency range of interest, in agreement with Stewart et al. [27], who concluded these were suitable phantoms in terms of steady-state mechanical properties. As attenuation is derived mainly from an absorption component and a scattering component, the macroscopic scattering properties indicate that these materials are suitable for preliminary studies of QUS characterisation, even though the accuracy of the underlying tissue microstructures as models of human brain tissue may be limited.

### QUS parametric images

**Fig. 4:**
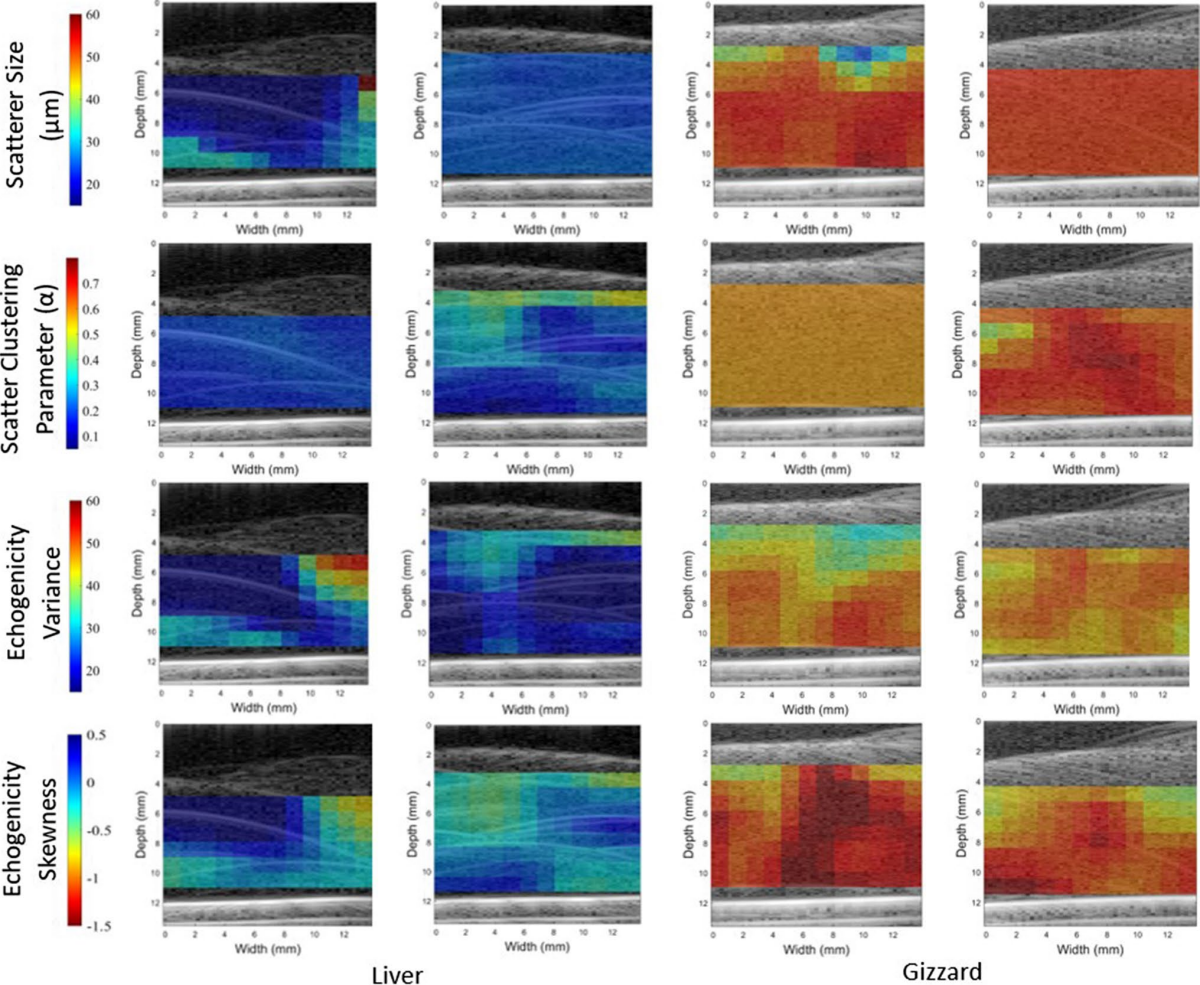
QUS images from two samples of both liver and gizzard. ROIs are selected from the original image, and QUS parameters are calculated and displayed and registered over the B-mode image. Parameters shown are scatterer size, scatter clustering parameter, echogenicity variance, and skewness, with the colour bar based on maximum and minimum values for all tissue types. The images show that all of these parameters exhibit classification potential, but there are still some outlying pixel results for some parameters

QUS parametric images indicative of the size of acoustic scatterers provide a fast way of distinction between healthy and cancerous tissues [28]. In this study, we proposed to use QUS parametric B-mode images as a differentiation method. Those images were generated from B-mode images with a colour overlay (Fig. 4). The overlay of colour was formed by assigning each pixel a colour based on the parameter value in that ROI, with reasonable colour bar axes. These images were produced for all thirteen QUS parameters for all samples. Examples of four parametric images for two samples of liver and gizzard are displayed in Fig. 4. The images highlight the significant differences in the parameter values for liver and gizzard for selected spectral, statistical and, echogenicity parameters. The scatterer size was found to be significantly higher in gizzard, suggesting that the scattering sources are larger agglomerations of scatterers, further evidenced by a higher clustering parameter. There was also a larger variance in echogenicity values in gizzard and a lower value of skewness of the echogenicity distribution. Although Fig. 4 also shows intra-sample variability between two gizzard samples, the colour coding in gizzard and liver shows huge potential for liver and gizzard differentiation using QUS parameters.

**Fig. 5:**
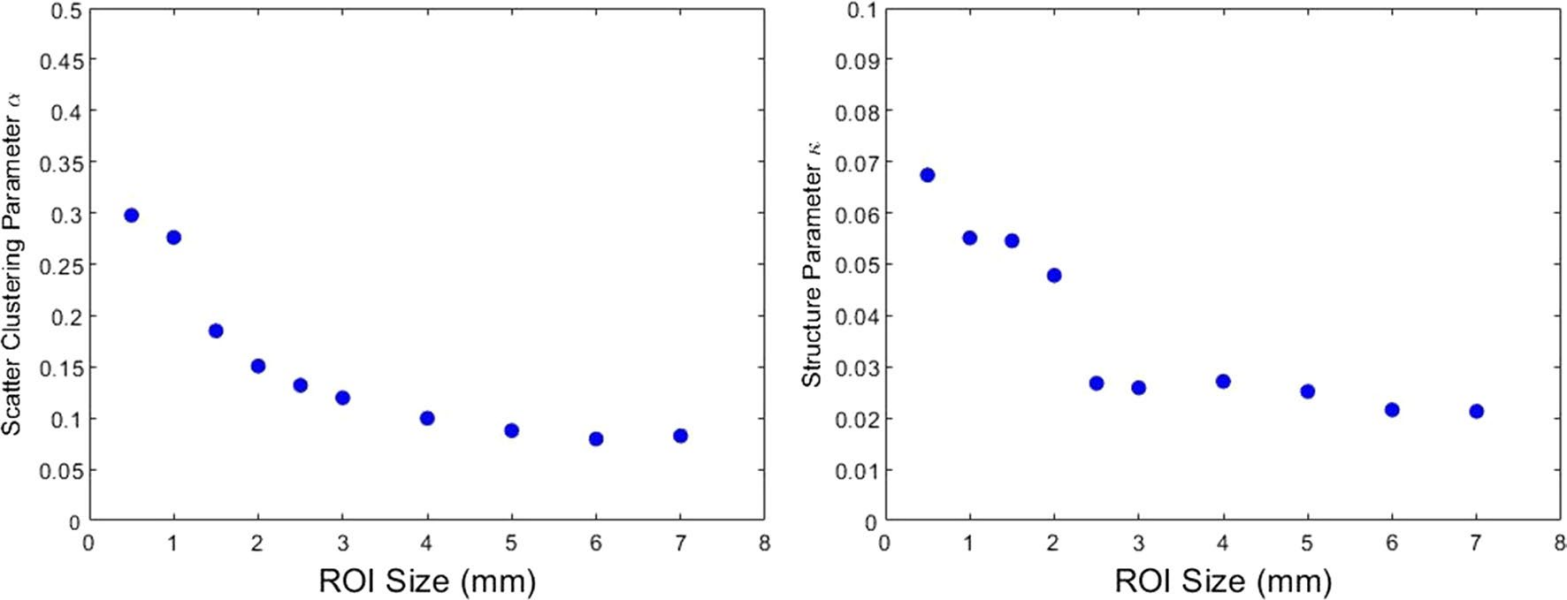
Effect of ROI size on homodyned K-distribution parameters

Whilst pixel values were seen to be fairly consistent throughout relatively large regions within each sample, the ‘edge effect’ was observed. This is an artefact of the sliding window method because the pixel values around the edge tend to be different to the bulk of the sample. It is particularly evident in the liver samples and could be reduced by using a larger window percentage overlap. There will always exist a trade-off between parametric image spatial resolution, computation time, and variance in statistical QUS measurements. The ideal window size, or gate length, has been considered in various studies of spectral parameters [29, 30], but there are fewer studies relating to homodyned K-distribution parameters. In the present study, a window size of 3 mm was used, which corresponds approximately to 10 times the wavelength of ultrasound in tissue at 5 MHz. The effect of ROI size on homodyned K-distribution parameters is demonstrated in Fig. 5 which shows the parameters tend to a limiting value at around 3 mm.

### Machine learning-based near-real-time QUS analysis

The computational cost is important if QUS is to be used as a real-time imaging tool for intraoperative tissue identification. For context, our analysis was conducted on a PC with the following specification: Windows 10 64-bit operating system, Intel Core i7-9700 K CPU @3.60 GHz, 16 GB RAM. The time taken to image a 1 cm × 1 cm region, similar to the images shown here, was calculated for the various parameter sets.

Echogenicity images were the quickest, with an average time of 4.7 s, followed by spectral parameters, which took 6.3 s. The algorithms involved in solving for parameters of the homodyned K-distribution require a tolerance value, which can be tuned to give more accurate or faster results. The tolerance value used in this study was 0.0001 for both *α* and *γ* estimations. This resulted in a total time for a 1 cm × 1 cm QUS image of 22 min.

**Fig. 6:**
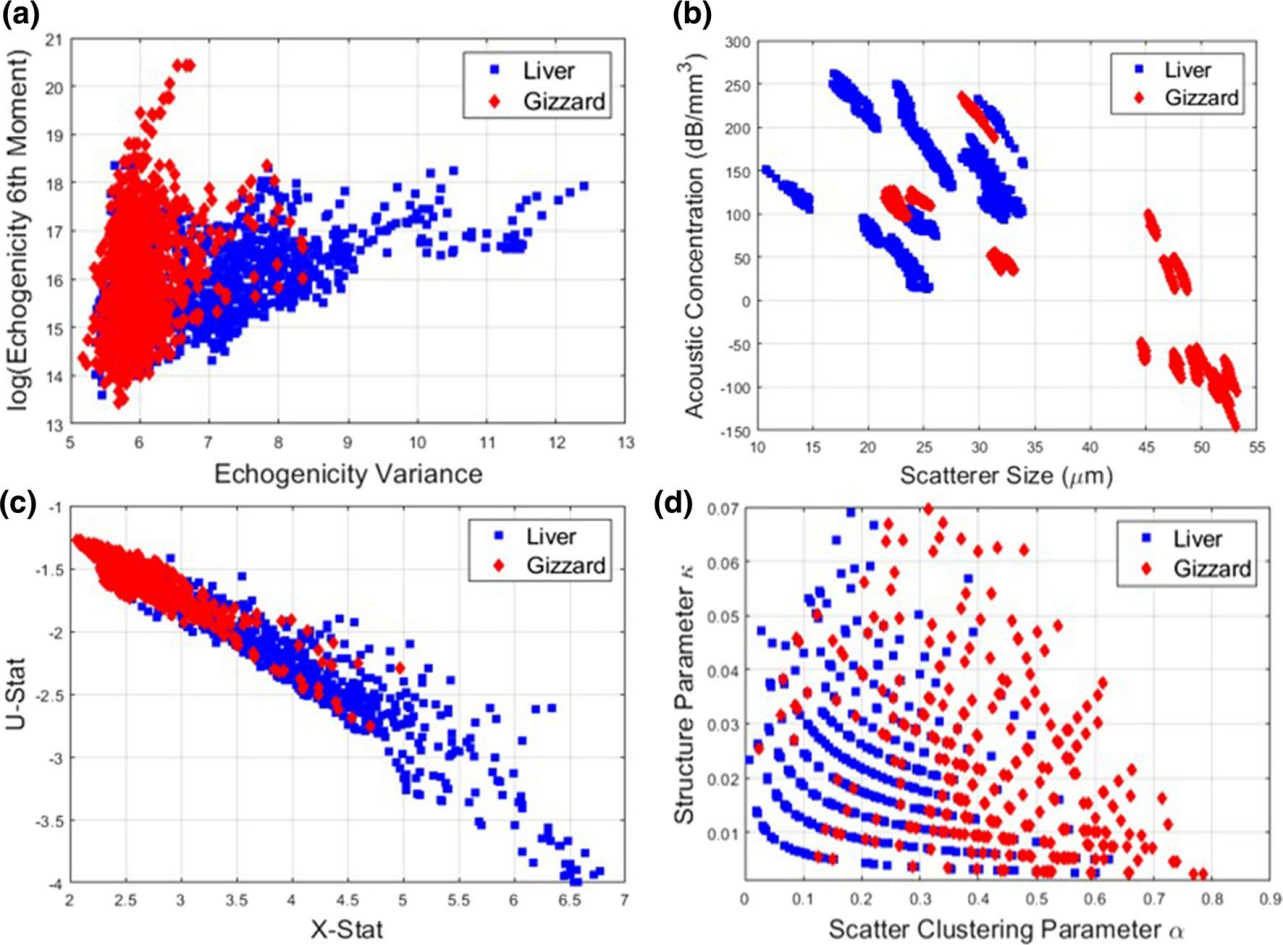
Results of QUS parameters. **a** Two echogenicity parameters showing distinct clustering of results for liver and gizzard. **b** Spectral parameter results highlighting the very small intra-sample variance. **c** The *U* and *X* statistic used to estimate the homodyned K-distribution parameters. **d** The results for scatter clustering parameter and structure parameter, with notable discretisation of results for *κ,* which may be due to the tolerance values of the algorithm

Figure 6 shows the numerical results for some QUS parameters for all 1 mm × 1 mm sections of tissue via the sliding window technique. One can observe the clustering of data, even when considering a 2-dimensional visualisation here. However, all correlations of those sample parameters show a degree of overlap that creates an uncertainty of applying those parameters to a clinic setup.

To provide rigorous evaluation of classification and explore a possible reduction in the number of parameters required achieving near-real-time QUS analysis, various combinations of QUS parameters were used to train a binary classifier based on an unsupervised machine learning method. The method chosen was the k-nearest neighbours algorithm, using five neighbours and validating over sixfold. A sixfold cross-evaluation of test data was calculated using a custom MATLAB function. In sixfold cross-validation, the dataset *T* is randomly divided into six equally sized (up to one instance) non-overlapping subsets *Ti*, called folds. For each fold *Ti*, a training set *Tri* is defined as *T* − *Ti*, the k-nearest neighbour classifier model *mi* is learned from *Tri*, and *mi*’s error is estimated on *Ti*. The mean of all these error estimates is returned as the final estimate.

**Fig. 7:**
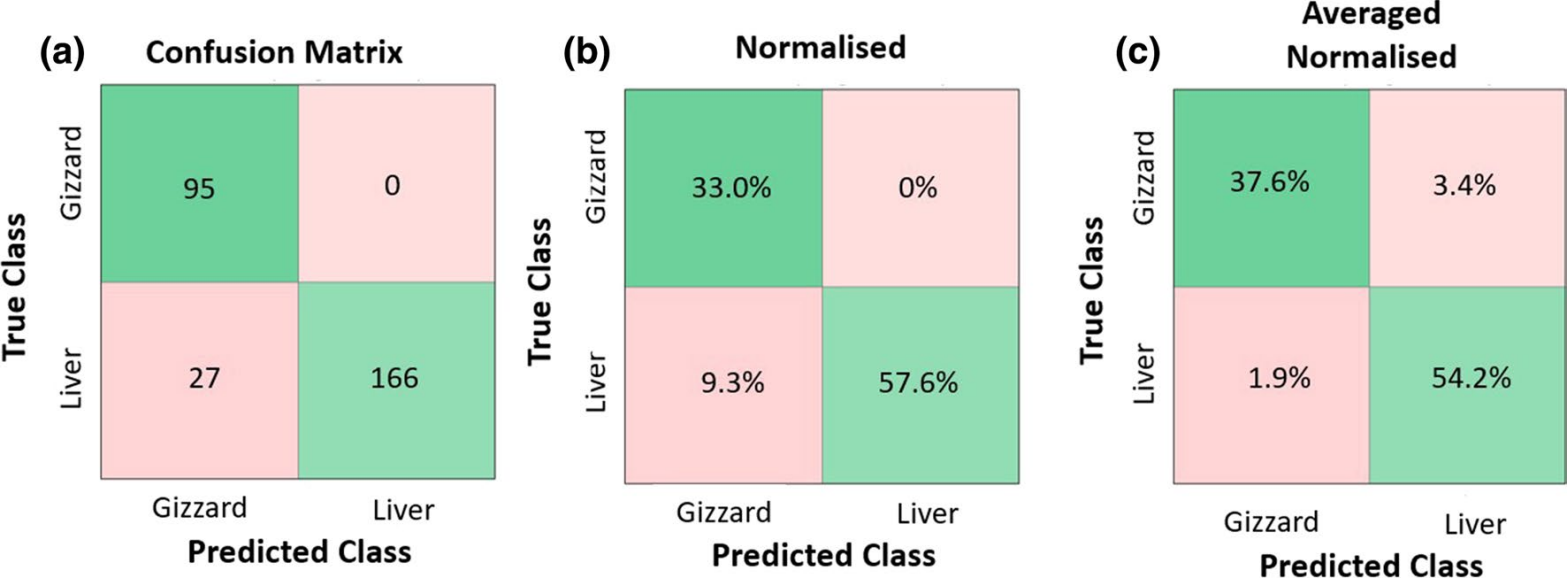
Results of confusion matrix. **a** Results from fold 1 of near-real-time parameters, **b** normalised results for fold 1, and **c** average normalised results of near-real-time parameters over all sixfold

The accuracy and F1-score for various parameter sets are shown in Table 3, with the typical confusion matrix shown for onefold, and the average of sixfold for the real-time parameters in Fig. 7.

**Table 3:** Sixfold cross evaluation results

	All parameters		Near-real time		Spectral		Statistical	
	Accuracy (%)	F1-score	Accuracy (%)	F1-score	Accuracy (%)	F1-score	Accuracy (%)	F1-score
Fold 1	90.3	0.870	90.6	0.876	79.9	0.767	80.5	0.762
Fold 2	97.9	0.974	97.9	0.974	61.8	0.525	85.1	0.814
Fold 3	96.3	0.951	92.0	0.887	79.3	0.642	66.3	0.760
Fold 4	96.7	0.956	97.7	0.970	80.7	0.678	71.0	0.664
Fold 5	95.4	0.940	93.5	0.914	82.4	0.725	84.3	0.819
Fold 6	96.9	0.968	97.8	0.979	95.7	0.957	67.3	0.639
Ave.	95.5	0.944	94.9	0.933	79.9	0.715	75.7	0.743

The result with the greatest individual accuracy came from the spectral parameters, followed by the statistical parameters, and then the echogenicity parameters. There is a remarkable increase when spectral parameters are combined with the other sets, giving an almost perfect classification. Interestingly, we did not see this improvement when only statistical and echogenicity parameters (nine parameters in total) were combined. The improvement when all three sets were combined was very small because of the already high performance of the combinations of two sets.

**Fig. 8:**
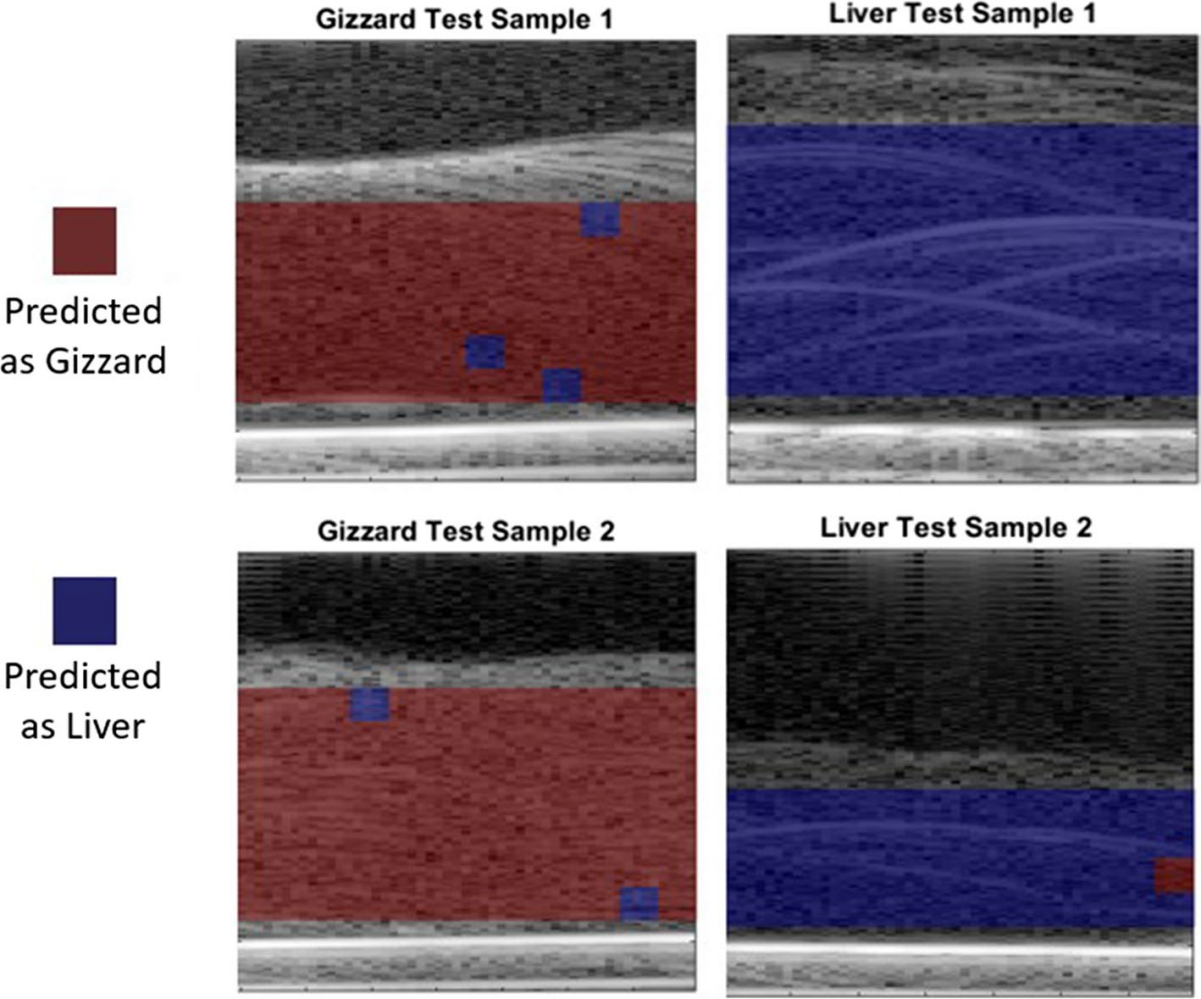
Prediction of liver or gizzard from classification algorithm based on echogenicity and spectral parameters from ten training samples chosen randomly. Binary results are superimposed on the B-mode image. The pixel resolution is 1 mm

In terms of computational cost, it would be optimal to use only spectral and echogenicity parameters while working towards real-time application with a total image formation time of 11.7 seconds. To illustrate visually how the near-real-time parameters performed, the predictions based on echogenicity and spectral methods were displayed over the testing samples. The capability of this technique to identify the two tissue types correctly can be seen clearly in Fig. 8. The accuracy of these particular four testing samples, which were randomly chosen, is 98%, with almost all of the incorrectly identified pixels located at the edges of the images. This problem could be overcome in future by using a larger window overlap or a higher frequency of ultrasound to allow more wavelengths within a smaller window to provide greater statistical accuracy.

A binary classification image like those shown could provide valuable information to surgeons in several seconds if a large set of training data could be collected from human brain tissue and tumour. While the resolution of this image is reduced to 1 mm from the original B-mode, the tools available to the neurosurgeon impose an accuracy of 2 mm in theory. For this application, the accuracy of the statistical estimates, or larger window sizes, is of more importance than final image resolution, which is suited to around 1 mm for surgical tools.

## Related work

The purpose of acoustic phantoms is to mimic the acoustic properties of fresh human tissue over an ultrasound frequency range of interest. Commercially available soft-tissue phantoms are produced following the International Electrotechnical Committee (IEC) standard guidelines of a speed of sound value of 1540 m/s and attenuation coefficient of 0.5 dB/cm/MHz [31]. However, the brain has been shown to have a slightly higher speed of sound and attenuation than typical soft tissue [9, 32, 33]. Recently, a 3D perfused brain-tissue phantom was constructed for ultrasound thermal therapy and imaging, which had a speed of sound of 1545 ms^−1^ and an attenuation coefficient of 0.74 dB/cm/MHz measured over the range 1–2.5 MHz [34]. Further progress towards a suitable brain tumour phantom came when a study tested the mechanical characteristics of agarose and hydrogel-based phantoms to determine suitable mechanical phantoms for brain and brain tumour and found chicken liver and gizzard muscle to be most suitable in terms of steady-state moduli [27].

## Discussion

With regard to future work, the present study made the assumption that a good macroscopic ultrasound phantom corresponds to a good QUS phantom. A further study provided evidence of using those parameters for characterising brain and brain tumour tissue [35]. However, due to the difficulty of obtaining human fresh brain and brain tumour samples, the effect of brain and brain tumour tissue’s inhomogeneity in QUS parameters is still unknown. Nevertheless, a framework has been demonstrated that can classify phantom materials using a research ultrasound system, with encouraging results. Further testing of this technique will come from in vivo and ex vivo measurements of human brain and brain tumour tissue. If a large data set can be accessed, a tool to identify tumour boundaries in neurosurgery could be developed in the future.

To further reduce the processing data of classification, we also intend to explore a method for supplying unprocessed RF ultrasound data directly into a supervised deep-learning system, such as a convolutional neural network (CNN) without QUS parameter calculation.

## Conclusion

There is a need for a real-time imaging tool to aid neurosurgeons in complete removal of brain tumours. Many brain tumours, particularly gliomas, are extremely difficult to resect fully because of their infiltrative nature and similarity to healthy brain tissue [35]. This study determined the acoustic properties of chicken liver and gizzard muscle and, through comparison with data from the literature, showed that these tissues were suitable phantoms for brain and brain tumour, respectively, in terms of density, speed of sound, and attenuation. Subsequently, QUS parametric images were formed using a sliding window technique. These showed promise for tissue differentiation, with parameters such as the scatterer diameter and the scatter clustering parameter showing significant differences in liver and gizzard. Further value from QUS data was demonstrated when parameters were combined to train a machine learning classifier. This gave almost perfect classification results while using only nine out of the total of 13 QUS parameters, which achieved near-real-time classification for clinic settings. In terms of diagnostic value and real-time optimisation, the combination of echogenicity and spectral parameters reduced the computation time to 6.3 s, while still achieving high classification accuracy for our ultimate goal of real-time tissue differentiation.
